# U.S. Truth, Racial Healing, and Transformation Funders' Briefing Program, January 19, 2021

**DOI:** 10.1089/heq.2021.29006.trht

**Published:** 2021-09-21

**Authors:** Gail C. Christopher, La Quen Náay Liz Medicine Crow, Melanie Greenberg, La June Montgomery Tabron, Paul Zeitz, Antti Pentikainen, José A. Rico, Tia Brown McNair, Angela Waters Austin, Charles Chavis, Isaiah Oliver, Diane Hosey, Grace Hou, Andreas Hipple, Srikanth (Srik) Gopal, Marcus Anthony Hunter, Hannah Adamson

**Affiliations:** ^1^ Co-Chair, USTRHT, Washington, District of Columbia, USA.; ^2^ Executive Director, National Collaborative for Health Equity, Washington, District of Columbia, USA.; ^3^ President and CEO, First Alaskans Institute, Anchorage, Alaska, USA.; ^4^ Managing Director, Humanity United, Washington, District of Columbia, USA.; ^5^ President and CEO, W.K. Kellogg Foundation, East Battle Creek, Michigan, USA.; ^6^ Convener of the National Coalition, USTRHT, Washington, District of Columbia, USA.; ^7^ Senior Advocacy Director, #breathewithme, Washington, District of Columbia, USA.; ^8^ Revolution Executive Director, Build A Movement 2022, Potomac, Maryland, USA.; ^9^ Secretary, USTRHT, Washington, District of Columbia, USA.; ^10^ Director, Mary Hoch Center for Reconciliation, George Mason University, Fairfax, Virginia, USA.; ^11^ Executive Director, Truth, Racial Healing and Transformation Greater Chicago, Chicago, Illinois, USA.; ^12^ Vice President, Office of Diversity, Equity, and Student Success, Washington, District of Columbia, USA.; ^13^ Executive Director, TRHT Campus Centers, Association of American Colleges & Universities (AAC&U), Washington, District of Columbia, USA.; ^14^ Founder, President, and CEO, One Love Global, Lansing, Michigan, USA.; ^15^ Maryland Lynching Truth and Reconciliation Commission, Arlington, Virginia, USA.; ^16^ President and CEO, Community Foundation of Greater Flint, Flint, Michigan, USA.; ^17^ Philanthropic Outreach, Embrey Family Foundation, Dallas, Texas, USA.; ^18^ Secretary, Illinois Department of Human Services, Chicago, Illinois, USA.; ^19^ Executive Director, Better Way Foundation, St. Paul, Minnesota, USA.; ^20^ Managing Partner, Humanity United, Washington, District of Columbia, USA.; ^21^ Professor, UCLA Department of African American Studies, Los Angeles, California, USA.; ^22^ Student, George Mason University, Fairfax, Virginia, USA.

## Opening Remarks

***Moderator:*** Gail C. Christopher

**Participants:** La June Montgomery Tabron, Paul Zeitz, and Antti Pentikainen

***Dr. Christopher:* Welcome, everyone, to this briefing. We are honored to have La June Montgomery Tabron with us for just a few minutes. La June, I know W.K. Kellogg Foundation has been embedded, has led this work for decades. When you launched Truth, Racial Healing, and Transformation (TRHT), you reached out and had philanthropic partners in the local community. So I wonder whether you could tell us why you think that is important.**

**Ms. Tabron:** Thank you, Gail, and I do want to welcome everyone to this call as well. We are excited that there are other funders interested in this work and wanting to partner in this work. Today is our National Day of Racial Healing, and I am looking forward to the dialogue today and the continued dialogue. And what I will tell you is: We know that there are many people in communities that want to do this work. They are eager to do this work. And just this week, we have had over 400,000 people access our website to download the tools, the resource guides, and the information related to racial healing. So, I want to tell you that this is a very urgent need, a palpable need, right now, and people are coming into the movement. When we created this concept, first of all, we worked with partners. This was not anything that was created just within the walls of the W.K. Kellogg Foundation. We wanted to support the efforts that were happening on the ground, and through those partnerships, what we know is this work is bigger than any one foundation, but it needs to be seated in community. And we knew that in order for the work to thrive in communities, we needed many partners, and this work could not be owned by an entity or even a particular space. We knew it needed to be nationwide, it needed to be a movement. And the movement is growing, and our show, the National Day of Racial Healing program today, will show you that this work is happening on the ground throughout the nation.

***Dr. Christopher:* OK. And it is. And we are very excited with the support of possibly congress, and maybe even the administration. Who knew our country would be so shattered, and it would require some real in-depth healing? But one quick question, La June: People think this work does not lead to concrete outcomes, to policy changes. Could you speak of that very briefly?**

**Ms. Tabron:** Yes. Again, on our program today you will see how communities have taken this work and made changes in their communities. One particular community, Kalamazoo, will talk about how they used the pillars of the TRHT, one of which was housing, and separation, and they have since changed legislation in their community as it relates to housing policy. So, the work is real, and again, it starts with truth telling. And what you will find is when communities have come together and faced their truths, but they have been very inclusive and how they have allowed stories to unfold around what is happening in their communities, what we see is great outcomes. You will hear from Buffalo, talking about how they approached workforce development in their community and changed, again, policies and work with corporate America to make sure that people do have the opportunities. So people hear “healing,” and what we know is “healing” is a very difficult word, but more importantly, it gets to system change work, and that is where the work really hits the ground, and that is where we see the transformation happening. And it is real. It is happening all over the nation, and as Gail mentioned, we hope it will happen at the federal level as well. And there is great progress going on in that regard, as well.

***Dr. Christopher:* La June, I know you have a full plate on this Fifth Annual National Day of Racial Healing, so please accept my heartfelt appreciation for taking just a few moments to share your sentiments and the sentiments of the foundation as we move forward in our briefing today.**

**Ms. Tabron:** Thank you so much, Gail, for inviting me. And as I say, this is the real work that has to happen. Racial healing for us is at the heart of racial equity, and you cannot skip the healing work. So, thank you for your leadership, Gail, and thank you, all the funders, for joining, and let us hope that there is a lot of work we can do together in the future.

***Dr. Christopher:* And I want to also—she mentioned it, but at the W.K. Kellogg Foundation website, they will be commemorating this day all day, and it will be available, and you will be hearing from many of the local partners. So, this lets us know—she let us know—something about the scope and the scale of the work. From the very beginning, we reached out to local philanthropic associations, we reached out to community foundations, to private foundations, family foundations, because foundations should be critical catalysts for this work around the country. Philanthropy means, literally, love of humanity, and we need more expressions of love of humanity in these critical times. I am going to invite my colleagues Antti and Paul to come in now and just give another insight into the national effort. Paul and Antti?**

**Dr. Zeitz:** Thank you, Gail—and thank you, everyone. I am truly honored and humbled to be part of this community and to have this opportunity to share some thoughts with you today. As we heard already, a grassroots community local movement is already under way after a decade of work by the Kellogg Foundation and by many other agencies and entities supporting TRHT and other racial justice and equity efforts. And building on those efforts, we have the opportunity and responsibility to strengthen a blossoming U.S. movement that is poised to really accelerate the journey to lasting racial justice, equity, and healing. And we are building a TRHT movement in the United States at the national, state, and local levels. Building on all that work, Gail and Marcus, who you will hear from later, and others worked with Congresswoman Barbara Lee over a couple-year period. And in the wake of the George Floyd murder, she decided on June 4 to introduce H.Con.Res. 100, which was a resolution calling for the formation of a U.S. Truth, Racial Healing, and Transformation Commission. She introduced that with 112 cosponsors, including representatives of the Congressional Black Caucus, the Hispanic Caucus, the Native American and Indigenous Caucus, Asian Americans, and a broad range of leaders in the congress. And at that time, Shelly Marc, her deputy chief of staff, asked all of us—myself and others, who are doing advocacy work—to form a coalition to really get behind the resolution. And over the 6 months of last year, from June until December, we were able to mobilize 170 cosponsors of that resolution in the House of Representatives. At the same time, Congresswoman Lee had brokered a partnership in the Senate with then-Senator Kamala Harris, and she had studied and reviewed and had agreed to introduce a sister resolution in the Senate. When she was selected to become the vice-presidential nominee, she passed the baton to Senator Cory Booker, and he introduced a sister resolution in the Senate, S.Con.Res. 50, in early December, and within a short amount of time there were 13 Senators who had joined on before the session ended in the 116th Congress. At the same time, we have been building the movement. We now have over 229 organizations that are supporting this call for a national commission. This includes many of you, who are involved with local TRHT initiatives, universities, historically Black colleges and universities, civic groups such as the NAACP Leadership Conference and Cobra, faith groups such as Sojourners, the Religious Action Center; and a broad range of youth activists as well, including March For Our Lives, joined the movement during the fall of past year. We are also mobilizing celebrities and artists through the Black Music Action Coalition and the #breathewithme Revolution, which is where I work on their leadership team. Just yesterday, we released a video on Now This (Now This Politics' Instagram page) with Alicia Keys and Mary J. Blige and many other top artists calling for the Biden–Harris Administration to launch a commission in their first 100 days. I would say this group—the leadership group that you will be hearing from—has had successful interactions with the Biden–Harris transition team, and we are hopeful that this moment that everyone is aware of will—that this is an idea whose time has come, it is urgently needed to ensure longstanding racial equity in our country. There are lots more to say about our work, but I will pass it on to Antti now. Thank you.

**Mr. Pentikainen:** Thank you, Paul, and I want to say how honored I have been to work with you and Dr. Gail Christopher. Gail has had incredible wisdom and contribution, and you, Paul, have done incredible work. I think the video that is just released yesterday must have gotten more than a million views at this point. I also want to recognize in the call my previous boss, Nancy Lindberg from U.S. Institute of Peace, when we both were there, and also my current boss, Alpaslan Özerdem, who is the dean of the Carter School (for Peace and Conflict Resolution) at George Mason University. I lead a center at the Carter School that is studying how to make these processes more effective in assisting those who are leading the designing of these processes, and I have just three points I want to make from that perspective. One is that those historical moments when these processes become possible are rare, and most often those opportunities are lost, so it is highly timely for this nation and everybody who wants to back this to get organized and to support those efforts. My second point is that processes that are focusing on commissions and commission reports fail to deliver transformative change, so what needs to happen in the nation throughout this country has to be planned on the onset. And my third point is that every community, every nation, and every context has to design this process based on their own language, their own history, and their own actors, and I think the United States is extremely fortunate to have had these efforts spearheaded by the Kellogg Foundation, but also the leadership by Gail Christopher, that it can build on a nationwide movement that is being done and work that is being done. And so, looking forward, finally, I am happy to share that the extent of these efforts—and Hannah, if you can show the maps—is nationwide. (Two maps side by side under the title Mapping of Current Initiatives: (1) U.S. Truth and Reconciliation Initiatives and (2) U.S. Truth, Racial Healing, and Transformation Initiatives). Besides these efforts that are being led at the campus centers, that Tia will later speak about in the next panel, we also have an emerging list of commissions, I think more than 40 local commissions, being initiated that quickly need support and learning to move forward. So that would be my number one suggestion to help them to learn from each other. And second, I do not think a national process in this country so wide and so complex can succeed without benefiting from knowledge that is working on the grassroots. So, I close here, and I hand it over to fellow leader, our incredible leader from First Alaskans Institute, Liz Medicine Crow, and thank you for also your leadership in showing and modeling in Alaska how this work can be built on in its practices and mainstreamed in these efforts. Over to you, Liz, to lead our first panel.

## Panel on Local Insights

***Moderator:*** La Quen Náay Liz Medicine Crow

**Participants:** José A. Rico, Tia Brown McNair, Angela Waters Austin, and Charles Chavis

***Ms. Liz Medicine Crow: Gunalchéesh* (“Thank you” in Tlingit)! How are you? Thank you so much. Good morning, everyone. In my Tlingit language, we say: *Yak'éi Ts'ootaat!* Good morning! In my Haida language, we say: *Sángaay láagang***, **good day. I am Tlingit and Haida, and I come from the community of Keex Kwaan (Kake), which is in the heart of southeast Alaska, in the heart of the Tongass National Rainforest. And I am so honored to be able to spend a few moments with you today. But I am really here to introduce to you some incredible leaders, and I want to get right to it. I would like to introduce José Rico, who is executive director for the Truth, Racial Healing and Transformation Greater Chicago. José brings the concept of solidarity between Black and Brown and all communities to life in Chicago as they partner to implement the TRHT strategy. I would also like to introduce Dr. Tia Brown McNair, vice president in the Office of Diversity, Equity, and Student Success and the executive director for the Truth, Racial Healing, and Transformation Campus Centers at the Association of American Colleges & Universities (AAC&U) in Washington, DC. Tia is the driving force helping to creatively actualize the vision for transformation and racial healing on college campuses across America. I would also love to introduce to you one of my fellow place leads—and I am so excited that I get to see her and hear her today—Angela Waters Austin, who is the president and chief executive officer of One Love Global, and the founder, producer, and host of *Equity Equals* radio show across Michigan. Angela embodies the idea of success against the odds in implementing a comprehensive TRHT strategy in the capital city of Lansing, Michigan. And of course, Dr. Charles Chavis, cochair of USTRHT, vice-chair of the Maryland Lynching Truth and Reconciliation Commission, assistant professor at George Mason University. Charles is the local leader in Maryland and here at George Mason University. He is courageously combining academic excellence with community organizing to bring truth about historic lynching to the work of racial healing and achieving justice. And more about them are in your briefing program, but I just wanted to warmly welcome all the panelists to our first panel, which is really focusing on local insights. The question that I have for our panelists to get us going is: What has changed as a result of this work? How do you know this? And what does it mean for your community?**

**Mr. Rico:** Thank you, Liz. Three years ago, when we started this work here in Chicago, we convened dozens of community-based organizations to help us design how this was going to look here in the city, and we got a good response from several hundred leaders. We trained about 40 racial healing practitioners; came up with a plan of how we wanted to make the changes in policy, in the economy, in some of the racial justice reform efforts that were very important for us here in Chicago; and we started doing about 30 circles per year to get people to understand the methodology but also what our transformation agenda would be. We were on a really good path: the metropolitan area is about eight million people, and hundreds of institutions were doing this work, so we got a good start. Now, what you see is that we see the four different sectors in our community really have adopted the TRHT framework in our city. We have hundreds and thousands, hundreds of community-based organizations throughout the city that are being part of the racial healing circles. We have thousands of individuals who this year, even though the pandemic was on, virtually participated in racial healing circles. Today, we have—a thousand people logged on the National Day of Racial Healing from 10 am to 3 pm. And the only reason we could only do a thousand is because we have 200 racial healing practitioners doing live circles as we speak. We have the government sector, both the state launch, the multimillion-dollar Solidarity Heals program so people in all Illinois could be part of the TRHT framework, and the city also just launched—and next week is doing—a public policy conference called “Together We Heal” using the TRHT framework. I was in a panel presentation with Dr. Gail Christopher yesterday with 200 businesses here in Chicago that the United Way sponsored, and earlier today I was in another panel presentation with the Chicago Community Trust where the business community wants to raise $500 million on the Together We Rise corporate initiative to implement the transformation and the repair that is needed from the harm that an economy based on suffering has caused. So we have come a long way. People at this moment in time see the framework as something that is viable, and in a big city like Chicago, different places are implementing different parts of the framework, and I am really excited about how we bring it all together in a cohesively unified front.

**Dr. McNair:** Thank you. Good morning, everyone. I just want to say thank you to the planning committee and to the leadership of Dr. Gail Christopher who I admire so much for the visionary work that she is doing on the TRHT effort. I just want to say for us at AAC&U—and that is the Association of American Colleges & Universities—we know that change cannot be sustained without intentionality and accountability, and for us at AAC&U, we believe that lasting change is very focused and a priority for our students, the next generation of leaders, to be prepared, to be fully prepared, to be the strategic leaders and thinkers to dismantle the false belief in a hierarchy of human value, to build just and equitable communities. And that is our goal of the TRHT Campus Centers is to really work collaboratively with faculty, administrators, community partners, students, and community activists, to really focus on what it means to have just and equitable communities. And along with our President, Lynn Pasquerella, and myself, we are honored to serve as part of the design team for the national TRHT effort. And in 2017, AAC&U partnered with the very first Penn TRHT campus—and Liz, you were there, and thank you so much for being there, part of that effort. And we are working with them on visionary goals, but also action plans, comprehensive action plans, focused on the work that they need to do within the institution and in the communities to achieve our shared goals. We often say that TRHT, the work of TRHT does not replace existing diversity, equity, and inclusion initiatives at campuses, but it makes a deep connection with what we value at AAC&U as part of being liberally educated, that we are speaking across differences, the civility that we do not see as often as we need to within our country, the healing, the deep listening, the empathy, and the action. We now have over 28—we have 28 partner institutions working with us; that reaches hundreds and thousands of institutions across this country. And we are working with them to build—we have the centers. We have TRHT Campus Center institutes every year in June, where they come together. We have had over 108 institutions participate in that. Over 400 practitioners have been prepared to facilitate our racial healing circles. And what we are also doing is we have partnerships with our national evaluators at the summer institute who develop comprehensive assessment and evaluation plans, because as you have already heard from my colleagues this morning, we have to be focused on action, we have to be focused on accountability, and we have to be focused on change. So, I just want to say that we are developing resources and tools, as I close out. We are developing resources and tools with our higher education partners on stages of implementation: campus, client, and assessment tools based on the TRHT framework. All of our campuses are developing strategies for examining narrative about TRHT within their communities and at their institutions. So, we are very excited about this work, we are excited to be in partnership, we know that it is necessary, and we believe that it is important for change to happen. Thank you.

***Ms. Liz Medicine Crow:* Thanks so much, Dr. Tia. Next, we will hear from Angela Waters Austin, followed by Dr. Charles. Angela, good morning.**

**Ms. Austin:** Good morning, sister. Thank you so much. It is so good to see your face and hear your voice. And greetings to you all from the wintry land of Lansing, Michigan, where the National Guard is camped out. Really interesting times. So, as we talk about movement and racial healing, I heard Dr. Christopher say that we could not have anticipated this, but I believe that she did. I remember back in Carlsbad, California, there were a few words that I heard that I left feeling fully committed. One was *jettison*, and I knew that a Trekkie was before me, and I heard *movement*, and I am like, “Yeah, I'm all about the movement, obviously.” What you did not hear is that I am also a local, statewide, and national, a global leader and cofounder in Black Lives Matter, and so that is an orientation that I bring into this work that is really important in terms of the local context and how we approach the work. Another word that I heard was *calling*. I heard *love*. To have these words spoken in a room about policy—ultimately the goal is policy and structural transformation, which is why we are having conversations about legislation and doing that deep work to uproot and jettison the systems of White supremacy that have had us bound and that cause harm to every person. Black and Brown and Indigenous people are harmed by White supremacy, but we have got to know that we are all harmed, and that we all have room and opportunity and space to heal, and that is what I love so much about TRHT. When I first saw the TRHT framework, what leapt off the page is that it is an organizing framework, and that has been our approach in metro Lansing as well as through our partnerships across the state of Michigan with the other TRHT sites, and with the other organizers and organizations that do deep movement work and that do deep policy work. And what this has really been for us is an opportunity to bring the grassroots movement into space with the institutional and systemic work that we have been doing in our community for the better part of a decade. And so, building that trust, healing circles have allowed us to build trust and to create and hold space for people to hear stories and to share narratives and to understand that we all carry pain, we all carry trauma. And for us to understand what alliance can look like, to understand what relationship can look like, to really understand that we are the change that we want to see, that we are the actors and the agents of the transformation, so that beautiful space, and so we call our—we kicked off Beloved Community this week. And so for us, to be in the middle of what looks like an attempted coup and what, our community is feeling really unsafe right now, we have folks that are holding healing circles in this particular moment. People are afraid, they do not know what is coming next. And to be able to have this tool that we have lived into and practiced into for the past three and a half years, we were ready. We were ready to say, “Let's pull it together. Let's create a space to hold our people right now.” And that is the beauty of TRHT, and I think you have picked up already, just hearing a few stories, that no TRHT is alike, because it is driven by community. It is driven by the unique makeup, it is driven by the unique culture, by the unique systems, and leaders and people, because it is all rooted in our hearts and in our minds and what we actually bring into the workspace. And so that is where the transformation begins. It begins on the inside, we bring it together, and we work together and we really have deep tough conversations, and I just want to applaud the Kellogg Foundation for accepting our proposal, because we named it, that it has to start with the grassroots, we have to honor the work that has already been done, and instead of TRHT coming in as something new or an umbrella, we said, “Let's create a platform where we can elevate that work, and honor those grassroots leaders, and actually elevate their work so that they could actually be seen and heard and powerful at the tables where institutional leaders are so comfortable making the decisions and calling the shots. “So, three and a half years, we have transformed our community into one that understands how important it is to follow Black and Indigenous leadership. That is the foundation for any possibility and endless transformation. And I am so excited to share that deep work with you. That means leadership development, our youth, our institutional leaders, our faith community, every sector. But the difference is, in our community we flip it on its head. It is not top down; it is bottom up. It is about how institutional leaders like yourselves actually come into relationship and listen and learn and actually cogovern and cocreate with community. I am so excited to be here with you today.

***Ms. Liz Medicine Crow:* Thanks, Angela. That was beautiful. I appreciate hearing your voice, sister. Dr. Charles?**

**Dr. Chavis:** I am going to try to go behind those three amazing activist leaders. My name is Charles Chavis. As mentioned, I wear many hats, but one of the major projects I am focusing on is the work that I am doing as vice-chair of the Maryland Lynching Truth & Reconciliation Commission. It is the first state commission established in the United States dedicated to investigating racial terror lynchings that took place in the state and the country. And we worked extremely closely with members of the community, specifically in Maryland, throughout Maryland, around salvaging this history associated with the tragedy, the legacy of racial terror. And as we began to develop our framework and think about what type of commission we were going to be, something that I appreciated—I am so glad that I see a few people here that I recognize from Villanova University, where I attended the TRHT training—I was really drawn in by the narrative change piece of this. And as I went back to the state commission, I introduced them to the framework, and they were sold. And I think, for me, as someone who works directly with descendants of racial terror lynchings and victims, and these families, the truth-telling is so central, and we have to get that full information at the forefront of this work. As a historian, I see that White supremacy is allowed to thrive because of the suppression of narratives. And the narratives are suppressed, whether it is marginalized or communities that have been overlooked, and a lot of these things are given a license to continue and go on. And so, again, I think, for me and the work that we are doing on the ground, we value the focus on the truth-telling at the local level and the narrative change work that is being done. And the way I describe how we are implementing this is through our work around preserving records and materials associated with cases of racial terror lynchings. Most recently we received a $300,000 grant from the Department of Justice Emmett Till Cold Case Investigations Program, and using the framework, outlining in our grant application, we were able to get funds to support the communities as they sought, as we investigated salvage materials and collected information regarding the cases, and also regarding the systemic inequalities that have persisted since these acts of racial terror took place. A hundred thousand dollars of those funds was actually set aside to support communities directly, and we are using that to establish a Historical Justice Fund that can support the communities that, to this day, as I mentioned, have not recovered. Where we see the highest percentages of lynchings taking place in Maryland, we actually see now, also, the direct correlations with People of Color and Indigenous people who are directly impacted disproportionately by COVID-19. We also see issues concerning juvenile justice and the prison-industrial complex, so many things that we know. In many ways, you can draw a direct line between the anti-Black racism and terror of old to the systemic inequalities that persist to this day in this country. And so, as we look to continue to work with communities in Maryland, we are so thankful for the leadership, thankful for this framework that has been tested by leaders and organizations, and that is really uniquely designed to fit the American context, because it is so important and is so valuable that we continue to engage at the local level with those who are directly impacted by this. To close, I think the piece around the social transformation, where we really appreciate about the framework in terms of the commission, is how we can use the narratives that we have salvaged, and then again to have conversations about what policy and structural change looks like, now that the truth has been laid bare for states and local communities. With that, I will turn back over to my sister, Liz. Thank you so much again for this opportunity.

***Ms. Liz Medicine Crow:* Yes. *Gunalchéesh*. I just really want to appreciate the stories of what is happening in the hearts of all of these communities of these states, these regions, across this country already, and what an immense opportunity to “level up” as a country and to utilize the hard work, the blistered hands, of so many across this country who are more than ready. One of the things that I would say is that—you know that saying that we have: “The sum is greater than the parts”? I think about TRHT as the parts and the sum are exponential. And we need to have an understanding that this is deeply rooted in community, and because it is deeply rooted in community, it will uplift the entire nation. *Gunalchéesh* (“Thank you” in Tlingit), *háw'aa* (“thank you” in Haida) to all of you and to our local leaders for taking time with us. As we know, everyone is so busy. *Gunalchéesh*, *háw'aa*.**

***Dr. Christopher:* Liz, thank you so much for moderating that panel. And I would be remiss if I did not ask you to say a little bit about the accountability partner approach that you have developed there in Alaska, as you have implemented with TRHT. Could you give us just a few words about that?**

***Ms. Liz Medicine Crow:* Yeah. Gunalchéesh, Gail. In Alaska, what we did was we took an approach of listening to our community about how and what they found value in in terms of moving forward, building from a place of aligned vision for what we wanted to see happen. And one of the things that we heard was—well, one of the important things, and this is kind of what helped us to create the accountability piece was—we heard from our people, and from people across the state, that we could not put government on a pedestal. We are working for policy change. Policy change is part of the system, and the system, we know, is extremely problematic. And that is what we are looking to transform. So, our community was saying to us: We need to speak our truth to our power, and we need to be able to have each other bear witness to our stories. And we do need government present, but we need them to be there in relation to community. Not to be the penultimate receiver of these testimonies and then ultimate determiner of our destinies. That model has not worked; it has only failed us. So, we need to speak to each other, right? And one of our agreements in our work is: In every chair a leader. So, we speak to each other. And we invite into that circle accountability partners, who are made up of government officials, institute agencies, departments, legislature, who are made up of church leaders, who are made up of business leaders, who are made up of community organizations and social groups, and who are made up of just people who want to see this change. And at the heart of what we ask our accountability partners to do is to be there to listen to these testimonies and to then look back at the legacy of their work, of their presence, of their place in Alaska, and to tell the story about that legacy after hearing these testimonies, what lens do they add, and then to sit again in that circle with all of us, for us to gather collectively to create the transformation, the solutions, and the policy work that we want to see moving forward.**

***Dr. Christopher:* Thank you so much. And I want to be mindful of our time, but I cannot express how my heart was moved by the stories that you all shared and how you have taken this concept and made it your own in your communities to really promote healing, so this is a blessing. So, thank you again, Liz, and all the others who represented the local communities. And they are just a small sample of many, many more. As Antti showed the map, this is going on all over this country. This is the news we *do not* hear about on any of the major networks, but this is the heart of America. We *can* heal, and I want to emphasize that.**

## Panel on Funders' Experiences

***Moderator:*** Gail C. Christopher

**Participants:** Isaiah Oliver, Diane Hosey, Grace Hou, and Andreas Hipple

***Dr. Christopher:* I have the privilege now of moderating the next panel, and this panel is going to be blessed with people who agreed to fund this work in its early stages and to be partners with local communities and be partners with the national foundation, the Kellogg Foundation, and I want to introduce our panelists. We have Isaiah Oliver, who came on board during the height of the Flint water crisis. Isaiah is the president and CEO of the Community Foundation of Greater Flint. We have Diane Hosey, who represents the Embrey (Family) Foundation. She is in Dallas, Texas, and they have done some amazing work there, and I would love to hear from them about the journey, what inspired you to do this work, and the accomplishments. We have two other very important panelists, and I am so happy that Grace Hou would join us from Illinois. She worked in the Woods Fund Chicago charitable foundation with us when we started the work in Chicago, and went on to be with the State of Illinois Department of Health and Human Services, and has carried this energy into her role there, and continued to be a funder of this work from that platform. And then finally, we are blessed to have Andreas Hipple of the Better Way Foundation, who I am meeting for the first time but I know from my colleagues that you continue to support the Indigenous communities around this country. So this panel is going to be an opportunity to hear from funders who have stepped into this work very early. My two questions to all of you—but I am going to ask Isaiah and Diane Hosey to go first, and then the final two panelists to follow: What inspired you to fund this work early on, before you heard stories like this from people who were actually doing the work? And what are some of the tremendous insights that you have gleaned from it that you think would be of value to your peers in philanthropy?**

**Mr. Oliver:** Thank you again for having me, and I am probably not appropriate for first, because if you remember our first engagement, I was a hard no! We were in the middle of the water crisis; I was really struggling with the work that we were doing. We were overburdened with the number of different pieces of work during that time, and I did not understand how much separation—the pillars of separation and the pillars of economy really played into the work that was happening in Flint. More specifically, the segregation and colonization and the concentrated poverty in the way they marginalized populations and majority minority cities like Flint were being impacted by structured inequality or inequities. And so, I did not understand that very well, and I had a lot of work to do, and I had just become president of the Community Foundation, and I was a hard no! This was back in 2017. Four years later, we are still engaged in the work, and very excited about many of the outcomes and many of the efforts that are happening right now. I like to say, very different than many philanthropies, I operate in a space where we engage people in the world of philanthropy. I work at a community foundation where we are soliciting folks to get their time, their talent, and their treasure, to make community a better place, and so in the early stages we were 1 of the first 14 place communities for TRHT through the Kellogg Foundation. And so we received a significant grant of $825,000 spread across 5 years to see this work implemented. And I could not go any further without thanking folks like Angela Waters Austin, who was really instrumental in the early stages of saying: We had to make sure this work is deeply rooted in community. Again, this was a community that, if we think about the timeline, had gone from—we are going from a water crisis to a national health pandemic to political polarization, and all this happening in that space to a global reckoning on race. And one thing that has been consistent is our work around TRHT. I could not be more thankful for the work, but what is offered by way of a framework or way to focus our work in ways that matter. So I will talk about a couple of things that have worked for us. One thing that has been important for us in this work has been our focus on making sure that we not only do work that is externally focused, but that we are also thoughtful about the history of philanthropy, not only in our community but also the history of philanthropy across this country and how it was created, and the inequities that it was built upon. If we are not thoughtful about that and turning the mirror on ourselves, we can really fall into some spaces that we have already fallen into in philanthropy and being top down and not engaging community appropriately. In the separation space, which is the space we spend a lot of our time in, over the past 4 years, we have trained many of our peers across the country, a number of folks who are healing practitioners, doing the community visioning process, or even bridging that community visioning process, to engage other folks in the conversations. Beyond that, we were really focused on our work with the city of Flint. One project that is amazing that I would love to lift up here is a project that we are working on with the Choice Neighborhood initiatives and our City of Flint Planning Department, where we were watching a community that was being transported from a public housing community that was moving from our far-south end of town to the north end of town. And there were lots of conversations about dialogue that needed to happen. If I am going to lean into the words of brother Martin Luther King, or Dr. Martin Luther King, who was honored just yesterday, our country honored him just yesterday, he would say that there is a process by which we “thingify” people in our communities. Or the process of thingification, where you justify thingifying a person; that way, you can exploit it, exploiting poverty. And what we have seen happen all too often is that process of thingification really lays into this hierarchy of human values, so you justify the thingification, and ultimately you see people as objects and not as people worth having conversations with. And the reality is, until we can start having more dialogue with one another, we are not going to be able to address the kernels of issues that exist in our community and really be able to move forward. So, we work very closely with the planning commission, with the Choice Neighborhoods initiative, with the City of Flint Planning Department, to really see to it that folks on the ground understood the connections, the humanity, of folks. Because when you dehumanize people, you really move to a space where you do not see them as peers; you do not see them as folks that you should work with. And so we have been working very closely, again, with groups to make sure that that happens. Another project that we have been working on very closely over the last year, as we think about COVID-19 and all that has happened with it, in our community we saw People of Color disproportionately impacted by the crisis. And when you see that happen, you have got to make sure that you have a multisectored group coming together to engage in conversation about how you address those issues, both the disparities or the differences in the numbers, and more specifically the inequities that perpetuate those disparities. And so we pulled together a multisectored group that had, many of them had been through the healing process. They had been through the healing circle. They really thought about this idea of prescription love as the way to address our community issues, and from there we built this model that is going to transition from a focus on our COVID-19 reality, our reality of the disparities with regard to COVID and COVID-19, to a process that is really going to be a racial equity roundtable focused on many of the issues that plague our community and how together with prescription love we can actually begin to address those issues. I will say finally the Community Foundation itself has anchored itself on equity as a tool of choice. And when you say equity is our tool of choice, we really began to look at both our internal and our external systems to see to it that we are focusing all of our efforts on making sure that people have everything they need to live and work and prosper. And at the core of that work is our TRHT work, and our partners in the TRHT space. So, thank you again for having me today, and I am excited about the conversation that we are going to pursue.

***Dr. Christopher:* Thank you, Isaiah. Powerful, powerful. Flint of all places, right? Let me invite Diane Hosey to talk about Dallas. I know you have had your own share of tragedies, one after the other, there. Please share with us some of your insights with your peers here in the philanthropic world.**

**Ms. Hosey:** Thank you so much, Gail. And Isaiah, you blew my hair back! We are in Dallas, Texas. We are in the south. It takes 3 min just to introduce ourselves! So I am going to do my best. Thank you. It is a privilege to be with all of you and to share a bit about the Embrey Family Foundation and our work and how our work is in transition. I have visual aids. I used to be a schoolteacher, so I bring visual aids. This is our logo (shows logo), the Embrey Family Foundation, and you can see it is very artistic, with our name kind of emerging out of this. Art as social justice is one of our key components, as well. We feel like art is inherent in the soul of who we are, in the soul of where we are at, and can lead the way very often. So, from Embrey Family Foundation to Dallas Faces Race, which was a forum that we invested in and created with another small family foundation, the Boone Family Foundation here in Dallas, and I will talk a little bit more about that, and how we got to Dallas Truth, Racial Healing and Transformation, and this, A New Community Vision for Dallas, which was released in 2019, the spring of 2019. Dallas Truth, Racial Healing and Transformation, I am proud to be on the steering committee, fund-raising chair. Do not ask me how that happened, but—One of the things I want to say, and in this report you will see that within a couple of pages it says: “Dallas is built on stolen land with stolen labor,” and this first visionary component, Jerry Hawkins, our executive director for Dallas Truth, Racial Healing & Transformation,—some of you know Jerry—is a historian by nature as well, and this document was filled with the history and the truth-telling of Dallas, which had been told in pockets but not widely, and of course that is the core component of healing, is tell the truth. The thing I would like to share with regard to our role in it is we had good leadership. Lauren Embrey came to the table and said, “We need to understand more about implicit bias. We need to understand more about racial equity.” She said all along that Dallas needed to have racial healing, and we started exploring how we do that. Through that, we put together a forum, the Dallas Faces Race, because we knew we needed to build capacity in the nonprofit community to start with, have a common language, have a common understanding, and really compel those nonprofits to start doing their own work to have the conversations with their board members, which was and still is a very challenging part of the process. Then, we had a robust group of folks join us. We brought in the Facing Race Conference in 2014, and a lot of our capacity building was to drive toward that conference. It was the largest attended in the south; they had never done it in the south before. And you all need to remember, doing this work in the south—that is all I need to say. It is—there is a lot to it. And nothing about it—it is not easy anywhere, but particularly, a lot of learnings, a lot of heartbreaks along the way, but we persist, right? And Gail, I have to say, you said, “Who inspires us?” Gail Christopher inspired us. Gail Christopher, we reached out to, shortly, and it was just synchronous, really, or the Dallas—Five Dallas police officers were shot and killed in July of 2016. People were coming to Dallas Faces Race. We had the only racial equity program officer in the city of Dallas at the time and saying, “What do we do? How is this happening?” And this occurred at a peaceful Black Lives Matter protest in 2016. We called, she responded, and said, “We're going to come. We're going to talk about truth, racial healing, and transformation.” We put the letter together with other foundations and said, “Please consider us for a grant.” The work has commenced. It is growing. We raised over $4 million in the summer and early fall. We know because of the Black Lives Matter protests and the crises that have occurred around that; many more funders are coming into the work than ever before in Dallas. So, we are encouraged by that. We are in “spend down” mode, and we are spending down by 2022. We are happy to share that story with anyone. We finally calculated, year to date, what we have invested in racial healing and driving toward racial justice in Dallas over 16 years: about $7.3 million, and that is been pretty much place based in north Texas and in Dallas. We are small. One of the learnings was if we had more money, more money would go into that. It took us a little bit longer, our learning curve was higher, so I would say: Learn faster and give higher right now. Thank you.

***Dr. Christopher:* Thank you very much, Diane. Grace, you want to tell us some more about Illinois and Chicago?**

**Ms. Hou:** Of course, I would love to. Good morning everyone, or good afternoon, everyone. It is so good to see you. Good to see you, Dr. Christopher, as always. It is always an amazing, for me, to be in a space, and I cherish the time that I can be with you, Dr. Christopher. And as she mentioned, I became a part of the TRHT journey when I was the president of Woods Fund Chicago, and I continued it as secretary of the Illinois Department of Human Services. So in many ways, I supported healing work as a private grantmaker, and then as a government entity, as well. And so, I think like Flint, and Dallas, we in Chicago were invited to be part of the first 14 places across the country to be a part of the W.K. Kellogg Foundation's TRHT work. And I think in terms of your question, why was I inspired, why do I continue to be inspired to do the work, it is because I have experienced it. After spending that initial week with thousands of colleagues across the country, I really understood how racial healing work could augment organizing: advocacy, policy change, and transformation. And I imagine most of the folks who we are today have probably already participated in a racial healing circle, but I would say that if you have not, then I would strongly recommend that you do it, and not any shortened version, but the full version, to really learn not only about others and the practice, but also about yourself. I carried that understanding, that priority of healing work, with me to my role as the secretary of the Department of Human Services and as part of Governor Pritzker's cabinet. And I cannot agree more with what Liz Medicine Crow has said. We need government to be present. And I am glad to say we are present here in Illinois, and under Governor Pritzker's leadership, each of our cabinet agencies had to develop a diversity equity and inclusion plan, and at our agency, the Department of Human Services—and we serve one in three people in Illinois—I feel that our work is to support humanity, that we are the heartbeat of the state. And so, the pursuit of racial justice is a critical piece of this, and healing. As I talk to funders, having been one myself, a government leader now that has the ability to—in some ways and in some ways not—move a lot of resources, if we want change, if our mission is to support better educational outcomes, or eliminate poverty, enable all to build wealth, if we want racial equity, I cannot see how we can do that without healing now. We cannot ignore the chasm that exists in so many different ways, and that has existed. And so to achieve everything that we set out in our mission statements and our vision statements, I feel like healing has to be a part of those objectives. We cannot skip this part. Our distance and racism were man-made. And so, we need to man-unmake it, right? And we cannot do that just with policy; it has to also be something that we are committed to. We have talked about how in 2020 we saw racial disparities magnified. It was always there, but it became larger in our own vision by the COVID-19 epidemic hitting Black and Brown communities the hardest. We also witnessed the fight for racial justice growing stronger and more visible as people came together in protest. And I think I and other leaders of the state recognize that it is impossible to do the work toward a better quality of life for people in Illinois without addressing the legacy of racism in our country and across Illinois. It is a time to be bold, right? We need to take action. And so we are doing that in so many ways across Illinois, leveraging federal investments, focusing on the hardest-hit zip codes, oftentimes Black and Brown communities, and rural communities. And one of the most amazing initiatives that we launched last year, which I am so proud of—perhaps the first of its kind led by a state—is we launched Healing Illinois, which is an initiative designed to fund racial healing activities in communities all across our very very diverse state. If you look at Illinois, and you think about us, we are blue, but we are blue and we are red. So, we have partnered with the team at the Chicago Community Trust to initiate this project, and it is working. We have awarded four and a half million dollars to nearly 200 nonprofit organizations and public agencies across the state to support racial healing activities that make sense for their communities. This is both inspiring and sad. We got more than double that in requests. What this is saying to us is that communities want to have these conversations; they need the resources to do it. These grants are funding activities such as listening circles for young adults (in Stephenson County?), Restorative Justice hubs for teens in Chicago's Austin community, mural projects, TRHT conversations, and then the development of diversity equity inclusion plans for organizations. That is just the tip of the iceberg. We are in this work with you, and I welcome you, for those of you who have time, to visit our work at (healing.illinois.gov). And I will turn it back to Dr. Christopher. Thank you so much for having me.

***Dr. Christopher:* Grace, thank you. And thank you for carrying the torch into government. That was brave of you, and we really do appreciate that. Andreas, I know that we are running on time, but please share with us your unique perspective in your funding of Native American and Indigenous groups.**

**Mr. Hipple:** Thanks, Dr. Christopher. I appreciate it, and I am honored to be here with you and with everyone. Better Way Foundation is based here in Dakota land, traditional Dakota land in Minnesota, and I am in St. Paul, but our offices are in Minneapolis, I am told. We are basically a smaller funder, a family foundation largely focused on education, but we realized, as we are now 5 years into a real strong focus on Indigenous communities, that truth and healing have to be a part of that. And I wanted to talk about pathways. I want to talk about what inspired us to get into this work, and it is our partners, it is the communities that we seek to serve as a funder, the communities that guide us into how to be a good funder and how to support meaningful change on their terms. Our focus on Indigenous education, particularly early and childhood education, has led us to do a lot of introspection about learning, learning about how catholic education, which has been a part of our framework historically, is littered with horrific violations of human rights, as well as opportunities now, today, to start taking responsibility and accountability for some of that history. As we entered into this work, our partners taught us about some of that history, and so we said, “We need to do our own homework.” And I think this is one of those pathways into this work: looking at our own programs, looking at our own work, and understanding where either the frameworks that we have had or the approaches we had had been steeped in White privilege and other elements of historic systems and White supremacist systems that we never thought of, perhaps, in the philanthropy that our foundation and others were doing for years and years. In that process, we learned more about boarding school history, and that led us to start funding the National Native American Boarding School Healing Coalition. I remember sitting in a session a couple of years ago in which Liz Medicine Crow and her team were presenting and continuing that learning process and understanding that we cannot fund an education without thinking about how to support healing. My good friend Trisha Moquino from Cochiti Pueblo in New Mexico says, “Truth and healing is so intertwined with language and culture for our Indigenous partners, for Indigenous people.” We cannot separate it. We cannot think about sovereignty. We cannot think about trauma and then (an acid-based?) approach to healing that has to be the way we think about how to support truth and healing in those communities. And so, truth and healing have become an important part of what we do because it is an essential part of listening to our partners, listening to communities. So the local-based approach that we are talking about today speaks truth to that, from my experience. And as a funder that is not the biggest funder in the room, is not the biggest funder in the country, we are looking for ways to really engage with our local communities here in Minnesota, in South Dakota, in New Mexico, where a lot of our work is based, and to do that, we need to be guided by them. And so, it matters how we approach that work. For us, more than two-thirds of our funding goes to BIPOC-led organizations—because it matters who you give your money to—organizations such as Red Cloud Indian School, which is a Jesuit institution that had a terrible boarding school history, but now is also willing to start digging into the archives, and they have created an executive director of truth and healing at that school who is now engaging with that community: Maka Black Elk, as an Oglala Lakota Nation citizen himself and a Jesuit-educated professional, brings the local perspective in his own community and is willing to do the personal work and listen to community and be led by community. I think an important part of this is we are not controlling what we fund. We are bringing our resources to help local processes go where they need to go, because ultimately change happens on the terms that community demands it, there is where it is going to have to be. Those of us in philanthropy, whether we are national funders or local funders, need to continue to listen and be guided by community. Finally, I want to talk briefly about that connection to the national (something?). From our partners in the Indigenous communities, we have seen incredible growth in engagement on the Truth and Healing Commission on Indian Boarding School Policy in the United States, an Act that Representative Deb Haaland from New Mexico cosponsored, incoming secretary of the Interior Deb Haaland. And that is the kind of work that can connect here as we think more broadly about different approaches to truth, healing, and transformation. My final word as a funder in this space is to not be afraid to make mistakes and not to be afraid to look at yourself. Myself as someone who is dripping with White male privilege, I have had to look at myself and do the work, and this is work that we can do. It is the opportunity to grow your impact; to really be transformative requires you to think, look at yourself as well. So, I encourage everyone to do that as well. Thank you.

***Dr. Christopher:* Thank you, Andreas. It has been amazing to hear from all of you as funders and to hear your heart connection to the primary of healing and truth, and bringing our communities together, in very respectful ways.**

## Questions

***Moderators:*** Melanie Greenberg and Gail C. Christopher

**Participants:** Hannah Adamson, Grace Hou, La Quen Náay Liz Medicine Crow, Charles Chavis, José A. Rico, Diane Hosey, and Angela Waters Austin

***Dr. Christopher:* I would like to invite Melanie Greenberg, who has agreed to facilitate Q&A, and again we would invite you all to put your questions in the chat and direct them to any of the panelists, the funders, the local leaders, any of us as part of a national team, and we have a good 10, maybe even 15, minutes to engage in some Q&A. So we hope to have that. Hannah, do you already have some questions?**

**Ms. Adamson:** Yes. There are already some questions in the chat. I see that we have one from Jimmy Biggs asking how TRHT works in racially and ethnically homogeneous communities, and how TRHT addresses extremist or supremist ideologies and behaviors, and whether there is an element of accountability and justice in this process.

***Dr. Christopher:* Well, those are three questions, but thank you! I guess I have to take that on, and I will also invite my colleagues who are doing the work locally. I will say that the work is about justice. It is about eradicating the fundamental permission to devalue some people more than others, and the framework for this work—it starts with narrative change; the second part is the healing, and the trust-building. But then we ask ourselves: How have systems of oppression been maintained for so long, and we realize that they have been maintained through systematic injustice in terms of separation, in many many myriad forms, but perhaps right at the center of that framework is the legal injustice, the justice that is embodied in the criminal justice system and its historic roots, as well as contemporary manifestations of that, in terms of voter suppression. So what you hear from the local efforts is that they work specifically on achieving lasting systems change work and justice work, but they do it by expanding the circles of engagement, so that they have the public and political will to achieve the necessary policy and systems change. And then the final piece of the framework is the economy. Ultimately the systems of oppression and exploitation, whether it was stealing land or enslaving people or exploiting immigrant workers, they were all about wealth, they were all about economics. And so White supremacy is the undergirding permission, if you will—although I always, I feel like our words have power. So, when you keep saying White supremacy, you put it in the air as if it is real, and it is a lie. White people are not superior, or supreme. And so we have to have better ways of creating a narrative that reminds us, ultimately, of our equality as human beings, so there is that. In terms of rural communities—or all-White organizations, for that matter—there is still diversity within those organizations, and there is diversity within those communities. They may be diversity of experience, they may be diversity of economic opportunity, diversity in terms of exploitation by the pharmaceutical industry. So wherever there is oppression and there is a need for justice, you can certainly apply this framework, and you can also connect the dots, help people to understand that they are part of a broader region, and they can begin to see their relationship with others. But I would invite any of the other colleagues, especially anybody who is doing work—I know the rural issue is an issue in Illinois, so Grace is still with us, she may want to answer that question, too.**

**Ms. Hou:** Hi. I would say that we are still waiting to see what that looks like in different communities across the state. We issued most of the grants at the end of past year, beginning of this year, so we are just beginning to see what that work looks like on the ground. I have to say that of the four and a half million, we did an allocation of how it should be distributed across the state, and one of the things that we did have a little bit more of a challenging time was to find organizations or entities that were interested in participating. But I am heartened to see that there indeed has been participation, and hopefully that will start a kind of energy and enthusiasm in rural parts of the state, because I do not think that this opportunity has been offered to them in the past.

***Dr. Christopher:* OK, that is good. Liz, did you want to add anything to that, about the rural?**

***Ms. Liz Medicine Crow:* Yeah. *Gunalchéesh*. I would say that one of the best things about being rural in Alaska is that we, despite differences that we may have in our communities, survive because of each other, and that is something that we place in the heart of the way that we approach the work, and that people have to have a good relationship, even where there is tension. So, we have used our shared Indigenous values to create a framework for how we engage. And especially when they are difficult and uncomfortable, we make a place for that, and we share something that our elders teach us, which is we only talk to you like this because we love you. And if we did not, we would not waste our breath. And so that places us in the center of a space of shared responsibility to each other, and it helps us move through those times when there are high tension points. I would say, too, that part of the question that I heard had to do with the extremists. And I would say this about extremists—and this is advice that we received from our visionary committee, which was: We have to think about this as a “pebble in the pond” movement, and at the center are the people who are there doing the work, and then those ripples reach out to others who are getting aligned and who are on their journey. And then through those, those kinds of systems of influence and connectivity, you start to reach further and further out. Well, one of the things that I think is really clear, especially recently, is that people who think they are on the right side have gotten a kind of free pass from showing up to practice. You know the saying: “You're only preaching to the choir”? Well, the choir has not been showing up to practice. As a result of that, we have what is happening in this country now. People who should have been pushing for these changes systemically and institutionally all along but were too uncomfortable made the decision not to actually take action, so it is not the extremists that really need to have their hearts and minds aligned; it is all the rest of the country, because the extremists will only be empowered by the people who do nothing to stop them from the job. And I think that that is an incredibly important place to see as we reflect on where we are in this country today.**

***Dr. Christopher:* Beautifully said, Thank you, Liz.**

**Dr. Chavis:** I also wanted to say something quickly about that as well, from looking at the establishment of the Maryland Lynching Commission. Something that I am extremely proud of in Maryland is that the commission was established as a result of the bipartisan unanimous vote in the state legislature, which was not an easy task, but it took working together with both Black and White community leaders throughout the state of Maryland to get this bill pushed through and done so in a unanimous and partisan way. We see this as being proof that it can be done both sides can come together around this work, but it takes time. But I think for me and our commission, we are extremely proud of that, and I think that that is one of the ways that we feel like this can be duplicated throughout the country, with this framework at the center. So those were just a few things I wanted to add onto it, as well.

***Dr. Christopher:* Thank you. Hannah, could you give us two questions? And then we will take our turns answering them. And Melanie, I am sorry, I am doing your job. Are you there, Melanie?**

***Ms. Greenberg:* You are doing a beautiful job, so continue.**

**Ms. Adamson:** Okay, so we do have a few more questions. I see Marc Gopin asks how the national movement will make sure that everything is elicited from the communities themselves, so that their own relationship building and healing capacities become the main driver of change. And also, just along with that, I see another question here. They ask how we can make sure that this work is in the front of the country challenging and inspiring our imaginations and actions every day.

***Dr. Christopher:* Two good questions. All right. I want to leave it to our panelists to respond to the first question from Marc. I mean, all of your presentations focused on community and how the community informed the work, but do you want to respond to that in another way? I think we know that we are only doing a national effort so as to continue to support and expand and integrate and build on your work. But any thoughts about how a national effort is informed by your work locally, which is obviously the work of community?**

**Mr. Rico:** Yeah, Dr. Christopher, if I could just speak briefly. Part of what we are doing here in Chicago is working directly with the groups that have been in the front lines of the racial justice work here in Chicago, and many of the public policy recommendations that they have been suggesting for years are the transformation initiatives that we are putting forward, whether—obviously—right now, the issues of the moratoriums on rents, the livability of people, the universal basic income, guaranteed access to public health, those are the issues, those are the public policy recommendations that we are promoting. We have three ways in which people could continue this work: this is by donating directly; for example, the youth groups that were in the front lines here in Chicago, Chi-Nations Youth, who has been on the front lines around the change in the narrative and the way in which Native Americans are depicted here in Chicago. Or a way in which people could get involved in the campaigns, how we could ensure that, for example, the state just passed the elimination of bail bonds here in the state of Illinois. A lot of that was done because of grassroots efforts by members of the Black community here in Chicago. And then also the education part. Many people here, particularly in the Black and Brown and Indigenous community, want to make sure that we look at the anti-Blackness that exists within our communities, the antiimmigrant sentiment that exists in the immigrant community, and how White Chicagoans could also look and educate themselves and their families in their communities around these efforts. So, these are very brief examples of how the grassroots movements that we are working with are those that are informing our transformation initiatives here, and we want to be able to make them and take them to the national level.

***Dr. Christopher:* Yes. Anybody else want to respond to that? The primary answer is by bringing people together. But I am making sure that their shared relationship, the things that people have illustrated all morning, in terms of when you bring these different groups together to hear it, to listen, and so when you think about a national movement, we think not only about our government but about the scope and scale of this work and building in ways to connect the dots and connect the people. The virtual capacity enhances that opportunity a bit, but definitely it is about weaving a whole cloth that reflects all of the folks. Solidarity heals! I love it. Thank you.**

**Ms. Hosey:** If I could jump in here—

**Ms. Austin:** I actually want to take on all three questions simultaneously. One, as philanthropy, you have an opportunity to be the champion to lead this work and to ignite it and seat it in your community. One Love Global, how did a grassroots leader gets into this mix of funders who are holding and convening this work? It is because none of the philanthropies in our community were willing to accept it and to make the commitment. It has meant that we had to create the foundation that we need, and so One Love Global is becoming what our community needs and what other communities across the state of Michigan—and we are finding across the country—need. So that is a very different orientation than one that we thought that we would have. And I just want to draw on the power of relationship in all of this, because it is only because we had relationships with folks because we think about extremism as being “out there” and “over there.” It is in us. It is in our families. It is all around us. And only because we had the relationship that we have cultivated over years that I have two moms who came to talk with me—Black Lives Matter Angela and One Love Global Angela, and they can see how I can embody those things in one body to talk about their sons and how their sons had been radicalized at the same time as they were embracing TRHT. And so holding healing in relationship has actually allowed us to expand from the urban area, and we have made this a regional effort, we are Metro Lansing, and so it is inclusive of our suburban families and our neighbors and our rural neighbors, and what is happening is that people are coming together across the entire region and learning how they can actually do this work at their dinner tables, at their churches, back in their communities where there are no Black and Brown people, and where they have actually succeeded in chasing out any diversity whenever it starts to rear its head. So this has been transformational, and that 4 years now, the institutions that would not even commit to hold the funding are trying to figure out how they can support TRHT.

***Dr. Christopher:* Oh, wonderful. Thank you, Angela. Yes. Michigan is certainly an epicenter for the challenge that we face as a nation. So, I really appreciate your voice. Other questions, Hannah?**

**Ms. Adamson:** We do have a few other questions. And I see one of them is especially about next steps and getting involved in what we need right now and moving forward, so I do not know whether you want to use that question now or use it to move to closing remarks as well.

***Dr. Christopher:* I will probably put that in the closing remarks. Let us try to take two of the questions. We have roughly 8 min left, so we can do two questions and then closing; that would be good.**

**Ms. Hosey:** I would just add with regard to the last question that being a small family foundation, we were used to really jumping quickly to move things forward, and I can tell you that I was, and we were frustrated with the amount of time it took to gather community and create the vision. And I even reached out one time to Rinku Sen (Executive Director, Narrative Initiative), and I said, “Oh my gosh, this is taking forever,” and she said, “You will regret it if you do not take the time to ensure that community is creating the vision for the city of Dallas.” And she was right, of course, and that is how the work continues. So I encourage the patience.

***Dr. Christopher:* Hannah, any other questions?**

**Ms. Adamson:** There aren't, and I would like to invite Melanie, if possible. I know she has some remarks from Humanity United and generally to share.

## Next Steps

***Moderators:*** Melanie Greenberg and Gail C. Christopher

**Participants:** Srikanth (Srik) Gopal and Marcus Anthony Hunter

***Ms. Greenberg:* I just want to thank all of you so deeply for the work you have done with the TRHT, and I have been so profoundly moved and inspired by everything that I have heard today, so I just want to thank you all so deeply for that. And thinking about where we move from here, I would like to put a proposal forward, which is understanding the role of philanthropy, in looking at our own narrative, at our own histories, and understanding that the power really needs to come from community, and to think about how funders can be helping in a real spirit of accompaniment. Could we start a more formal funders' forum that can live with this work, support this work, engage our own community in the kind of reflection, and healing and movement forward that we have all talked about today? And with that idea, I would love to introduce Srik (Srikanth) Gopal, who is the managing partner at Humanity United and has been a real champion for this work within an organization that has traditionally worked outside the United States. So Srik, I wonder whether you want to take just a moment before we move on to the rest of our wrap-up?**

**Mr. Gopal:** Yeah, thanks, Melanie. I just want to echo what Melanie said. Traditionally, we have always had a mission around enduring peace and freedom, and we have traditionally worked abroad in postconflict situations. And really over the past, I think, year or so, we have become keenly aware of the need to do this work here at home. And the fact that we need to have credibility as these builders at home to be able to work as these builders abroad. So we need to invite all the other funders on this call to examine how racial injustice really is the common fabric underneath all of the issues we work on, whether it be education or health care, community involvement, the justice system—really, under all of that as a common fabric of racial injustice. And until that fabric is mended, we would not be able to make significant and sustainable progress as a country. So really, I would invite the other funders, please join us in this effort, and look forward to the next steps around having a funder community on this.

***Dr. Christopher:* Wonderful. Thank you for that, and I hope folks will sign up for that, and I think we have a survey form where folks can complete that. I am going to invite Marcus Hunter, one of our coleaders of this national movement effort, to say some closing remarks.**

**Dr. Hunter:** Thank you, everybody. Greetings, beautiful people. I am Dr. Marcus Anthony Hunter. I have been with Gail and Mike (Michael Wenger, maybe?) at the call of Congresswoman Barbara Lee now some 4 years ago, working on the resolution, and also with Dr. Paul Zeitz who briefed with me as transformation architect. I am also a UCLA professor, and I love the sweatshirt #BlackLivesMatter. You are formally meeting the creator of the hashtag Black Lives Matter here, so I am happy to be present in that way. After a long Zoom call, I want to ask everyone to close your eyes very briefly while I share a proverb with us all from the great nation of Ghana, which, of course, is the place upon which many of us who are Black arrived in America. The proverb goes as following: “A lie can destroy a thousand truths.” I'll repeat it again. “A lie can destroy a thousand truths.” So that is from Ghana. And that wisdom is anchored in the work that we are up to, that—how many lives have been told, and how many thousands of truths deserve their time in the sun? We have been working as a collective, mostly all, on the love and on the volunteer of it all. And through that work, we have found a mighty coalition of folks from across the spectrum, so in many ways TRHT is also a movement of movement. We have Black Lives Matter represented. We have people who are about police reform represented. People who are interested in the livelihood of women and girls, Black women and girls, Black boys and girls, represented. We also have #breathewithme represented, which, just to give people a sense of the numbers of what we have been able to do, just from volunteering, the PSAs that we have been able to put out that feature activists and now musicians have seen over five million views. So, this is just off of the love and strength of a committed group of people. A hundred and seventy Congresspeople, representatives in the House. We have 15 Senators just in the Senate just off of the strength and love of what we have been doing together. I want to end with a proverb that is my own. I always ask, as a professor: If you hear it, you like it, remember I told it to you. And that is: Each of us needs all of us. Each of us needs all of us to think about how philanthropy can help make us more effective, how we can build up capacity. Each of us needs all of you to help do that work, to build our true capacity, so that what we see—the TRHT work—we do not have to look at it as missing components that could be there, that it is a comprehensive effective movement that has so many different avenues that what we are living in is a world where the idea of human hierarchy, the lie, has been replaced with the thousand truths that we already know, that the dispossession of labor and land, of Native and Indigenous and Black people, is a truth that we no longer allow to be covered by lies. I really am so grateful to everyone for your time and for your attention. As Dr. Christopher has mentioned, we have follow-up materials where we can answer more questions. We hope this begins a great dialogue, but please know that we need you. We need your continued commitment. We are grateful for the commitment that many of you have made locally, regionally, rurally, and urbanly, to this work, and this movement, like Nipsey Hussle says: The marathon must and does continue. I will pass it to Gail to close us out.

***Dr. Christopher:* Well, we are closed out. Thank you very much, Dr. Hunter. Honestly, thank you all for participating today. Our purpose was to give you a hint, to show you the scope and scale of this work, and to hopefully engage you in seeing the possibilities that are there. Again, we know philanthropy means love of humanity, and America right now needs a lot of love. And so, thank you. We are going to stick to our commitment to the time frame but do stay connected. We will be in touch with you. We will share our materials. And I hope that you respond to the invitation that we just heard to create a forum whereby funders can dig more deeply and share their insights and knowledge, as you heard from our funders' panel today. So, it is hard to applaud by Zoom, but I just want to applaud everyone that helped to make this happen, to honor all of our partnerships; George Mason University has been tremendous as a partner in making this happen. So thank you all, and have a blessed day. And we will have a peaceful transfer of power tomorrow in our inauguration ceremony. Thank you again.**

## Notes

This special publication is supported by the National Collaborative for Health Equity.

